# Association between infant feeding practices, COVID-19 related cognitive factors, and postpartum depression during the COVID-19 pandemic: a cross-sectional online study in Thailand

**DOI:** 10.1186/s12889-025-22672-w

**Published:** 2025-04-11

**Authors:** Wichukorn Suriyawongpaisal, Punpawee Kittikul, Eun Young Lee, Li-Yin Chien, Yan-Shing Chang, Kelly Pereira Coca, Doungjai Buntup, Seo Ah Hong

**Affiliations:** 1 ASEAN Institute for Health Development, Mahidol University, Nakhon Pathom, 73170 Thailand; 2 Breastfeeding Clinic Nakhon Pathom Hospital, Nakhon Pathom, Thailand; 3 Department of Nursing, Catholic Kkottongnae University, Chungcheongbuk-do, Republic of Korea; 4 Institute of Community Health Care, College of Nursing, National Yang Ming Chiao Tung University, Hsinchu City, Taiwan; 5 Florence Nightingale Faculty of Nursing, Midwifery & Palliative Care, King’s College London, SE1 8WA London, UK; 6 Escola Paulista de Enfermagem, Universidade Federal de São Paulo, São Paulo, Brazil; 7 Department of Community Health, Faculty of Public Health, Mahidol University, Bangkok, Thailand; 8 Department of Public Health Administration, Faculty of Public Health, Mahidol University, 420/1 Ratchawithi RD., Ratchathewi District, Bangkok, 10400 Bangkok, Thailand

**Keywords:** Postpartum depression, COVID-19 knowledge and attitude, COVID-19 vaccination, Infant feeding practices, Postpartum mothers

## Abstract

**Background:**

Maternal beliefs towards COVID-19 vaccine safety may be associated with infant feeding practice and postpartum depression (PPD). Since there is a paucity of studies, this study aims to identify associations of COVID-19-related cognitive factors (e.g., COVID-19-vaccination-related belief and COVID-19-related knowledge and attitude) with infant feeding practices and their associations with PPD during the COVID-19 pandemic in Thai postpartum mothers.

**Methods:**

A cross-sectional online survey was conducted among 840 postpartum mothers whose infants were less than or equal to 6 months old. Mothers with a score ≥ 13 using the Edinburgh Postnatal Depression Scale (EPDS) were considered to have PPD. Student t-tests were used to measure the association between infant feeding practices and COVID-19-related cognitive factors, and Chi-square tests were used to assess their associations with PPD. Significant variables (*p* < 0.05) from the chi-square test were included in the logistic regression analysis. Multivariable logistic regression analyses were performed to identify factors associated with PPD. The associations were reported in adjusted odds ratio at 95% confidence interval.

**Results:**

This study showed one-third of the participants (32.4%) were at risk of having PPD. Mothers who fed expressed breastmilk had positive beliefs towards COVID-19 vaccination and higher scores on COVID-19 knowledge and attitude, while mothers who fed infant formula or solid, semi-solid, or soft food had negative beliefs towards the vaccination compared to their counterparts. Multivariable logistic regression showed women who fed their infant with solid, semi-solid, and soft foods (AOR = 3.28; 95% CI = 1.35–10.92) had significantly higher odds of PPD. Among COVID-19-related cognitive factors, negative or moderate COVID-19-related attitudes were associated with higher odds of PPD (1.91; 1.19–3.07 and 1.85; 1.20–2.86, respectively). Socio-demographic factors associated with PPD included living in urban areas, residing outside the Southern region, having food insecurity during the pandemic, having unintended pregnancy, and having health problems during the perinatal period.

**Conclusions:**

The association of COVID-19-related cognitive factors and feeding practices with PPD may suggest that proper education on prevention, control, and vaccination of emerging infectious diseases such as COVID-19, as well as support for appropriate infant feeding practices, should be provided to postpartum mothers, which ultimately contributes to improving their mental health.

## Introduction

Postpartum depression (PPD) includes a myriad of symptoms that can occur to a mother after delivering her baby and may last for months [1]. The symptoms include loss of interest in daily activities, anxiety, an increase in negative thoughts, misery, fear of being able to raise a child properly, and, in some severe cases, thoughts of self- or infant harm. A recent meta-regression analysis study reported that 17.22% of the global population had experienced PPD, with population in low- and middle-income countries having a higher prevalence of PPD than those in high-income countries [2]. In Thailand, an upper middle-income country according to World Bank classification, the prevalence from a national study was reported to have increased from 8.4% in 2013 [3] to 16.8% in 2017 [4].

COVID-19 has had an unprecedented effect on health and well-being across the globe. Around 6.3 million deaths are estimated worldwide, with over 500 million infected cases [5]. This has forced many countries to implement public health measures such as lock-down, social distancing, vaccination, and wearing face masks, as recommended by the World Health Organization (WHO) [6]. In Thailand, around 4.5 million cases have been confirmed, with nearly 30,000 deaths as of 30th May 2022 [7]. Thailand faced a much worse scenario in 2021 when emerging clusters spread from the Central region of Thailand to other regions. At the end of the second quarter, Thailand had 259,301 accumulated infected cases, and this continued to rise dramatically throughout the year, with 1,069 deaths in June 2021 [5]. Thai government declared 45 provinces as ‘red zones,’ which restricted travel and working hours in some businesses, particularly in the service and entertainment sectors, and six provinces as ‘maroon zones,’ where onsite services by the catering and entertainment sectors were banned [8]. The COVID-19 prevention and control measures may disrupt social interaction and the daily activities of the general population, and it could lead to depression. Many studies reported an increased prevalence of PPD as a result of COVID-19 pandemic [9, 10] and PPD prevalence in developing countries had higher odds of increasing when compared with developed countries [10].

Previous studies reported that PPD was associated with COIVD-19-related cognitive factors (i.e., knowledge and attitude towards COVID-19 and infant feeding), and feeding practices [11–15]. Many studies reported mothers with depression were more likely to feed their infants with infant formula or other food sources rather than breastmilk [1, 11, 13, 16]. Mother’s fear of COVID-19 may influence their feeding practices [16, 17] directly or indirectly through cognitive factors, including beliefs about COVID-19 [14]. Studies showed that mothers with low levels of understanding about COVID-19 could switch from exclusive breastfeeding to other methods, such as expressed milk or a combination of breastfeeding and baby formula or baby formula to prevent the spread of COVID-19 to their infants [16, 17]. Recent studies from Bangladesh, China, and Thailand found that beliefs related to vaccine safety, including the notion that vaccines may affect breast milk, were factors influencing vaccine acceptance among lactating women [18–20] Yet, few studies exist on maternal beliefs about COVID-19 vaccination. Moreover, there are limited studies on the association of beliefs about COVID-19 vaccination with infant feeding practice and PPD.

Therefore, this study aims to determine the association between COVID-19-related cognitive factors, infant feeding practices, and PPD during the COVID-19 pandemic among postnatal women in Thailand. Understanding the context and associated factors will help health sectors in developing countries improve their services for postpartum mothers.

## Methods and materials

### Study design

A web-based cross-sectional study was conducted among postpartum women in Thailand from July to October 2021 as part of a multinational survey. The cross-sectional online survey was created using Google Forms. The survey link was distributed to Thai postpartum mothers through social media (such as Facebook, Line, and WhatsApp), personal social networks, websites, or posters in hospitals/clinics across various regions of Thailand. Mothers aged 18–49 years and up to 6 months postpartum were invited. A total of 919 participants completed the survey, and after excluding those who did not meet the inclusion criteria, 840 responses were included in the analysis. The questionnaire was initially developed in English, and all the investigators from the five participating countries reviewed, evaluated, and revised the questionnaire. The questionnaire was then translated into the languages ​​of each country, including Thai. Two health professionals assessed the Thai version of the questionnaire, which was then pre-tested with 20 postpartum mothers and revised for clarity. The study received ethical approval from the Institute of Population Science Research, Mahidol University (2021/03–042). The respondents were informed that their participation was voluntary, and an online informed consent form was provided and collected before survey initiation.

### Measure of variables

#### Postpartum depression (PPD)

The symptoms of PPD were assessed using the Edinburgh Postnatal Depression Scale (EPDS). The scale included 10 self-report questions based on individual experiences in the previous seven days. Various aspects of mental condition, including happiness, anxiety, sleep deprivation, sadness, and tendency for self-harm, were covered. This study employed a cut-off point for PPD at 13 or higher since the measure is valid for the Thailand context [3]. Participants who scored 13 and above were classified as PPD.

#### Infant feeding practices

Infant ages were grouped into less than 3 months and 3 months or above. The participants were asked, “How was your youngest baby fed in the last 24 hours?”. The question had four categories: (1) breastfeeding (yes or no), (2) expressed milk (yes or no), (3) formula milk (yes or no), and (4) solid, semi-solid, or soft food, including liquid (yes or no). The impact of COVID-19 on breastfeeding behaviors, including (1) directly on breast and (2) expressed breast milk, was assessed by asking, “Did COVID-19 affect your infant feeding behavior?” with the responses (i) not intend to feed, ii) shorter than I intended, iii) the same duration as I intended, and iv) longer than I intended). Timing of any solid, semi-solid, or soft foods was categorized into (i) not introduced yet, (ii) before 6 months of age, and (iii) 6 months of age or above.

### COVID-19-related cognitive factors

The COVID-19-related knowledge and attitude questions were adopted and modified from previous studies [15, 21]. Knowledge of COVID-19 included nine statements about transmissibility, symptoms, and prevention methods. Respondents were asked to choose an option (‘true’, ‘false’ or ‘do not know’) to the statement ‘COVID-19 CANNOT spread through the respiratory droplets of infected individuals. A correct answer was coded as ‘1’, indicating an ‘adequate answer,’ while an answer of ‘do not know’ or ‘false’ was coded as ‘0’, indicating an ‘inadequate answer.’ The participants’ responses to the nine statements were aggregated (0 to 9 scores). A higher score indicated a higher level of knowledge. The attitude towards COVID-19 was measured in terms of disease severity, preventability, and the importance of preventive measure compliance with seven statements (e.g., ‘Social distancing is important to prevent COVID-19’) with 5-point Likert scales (‘1 = strongly disagree’, ‘2 = disagree’, ‘3 = undecided’, ‘4 = agree’, and ‘5 = strongly agree’). The scores of the seven statements were aggregated (a total of 7–35 scores). A higher score indicated a more positive attitude.

Statements on beliefs about COVID-19 vaccination were developed based on the Institute of Global Health Innovation [22]. Four statements, for example, a COVID-19 vaccine is safe for breastfeeding women and babies” with 5-point Likert scales (‘1 = Strongly disagree’, ‘2 = disagree’, ‘3 = undecided’, ‘4 = agree’, and ‘5 = strongly agree’ were presented to mothers. Negative statements were reversely coded were reversely coded before summing. The total scores ranged from 4 to 20, and a higher score indicated more positive beliefs about breastfeeding.

### Sociodemographic factors

The sociodemographic factors included in the study were age, level of education (standard education was regarded as participants who had completed 12th grade or high school equivalent), employment status, marital status, family type, residential area (urban or rural), region (Bangkok, Central, North, Northeast, or South), and monthly household income during COVID-19 (< 15000 THB or ≥ 15000 THB). The impact of COVID-19 on food security was measured by asking, “Did you ever run out of food before the end of the month or cut down on the amount you ate to feed others?” divided into before COVID-19 (2019) and during COVID-19 (2000–2001) periods (response options = yes or no). The two variables were combined to categorize food insecurity as (i) food insecurity (insecure to insecure), (ii) worse (secure to insecure), (iii) better (insecure to secure), and (iv) food security (secure to secure). The variable was further categorized into (i) food insecurity, (ii) worse, and (iii) secure (insecure to secure or secure to secure) for analysis. Variables related to infants included low infant birthweight categorized into (i) less than 2.5 kg, (ii) 2.5 kg or higher, number of children, which was disaggregated into ‘one child’ and ‘two or more’, and birth interval grouped into (i) no siblings, (ii) less than 3 years, and (iii) 3 years or higher. Also, participants were asked if they had health problems during the perinatal period (i.e. yes or no).

### Data analysis

Statistical analyses were conducted using SPSS version 25 (IBM SPSS Statistics 25). Regarding descriptive statistics, the distributions of variables were presented as numbers and percentages for categorical variables and mean with their standard deviations (SDs) for continuous variables. Pearson’s chi-squared test or Student’s t-test was used to compare the proportions or means as appropriate. The logistic regression analysis included significant variables (*p* < 0.05) from the chi-square test. After confirming the absence of multicollinearity, multiple logistic regression analysis was performed to examine the factors associated with PPD. Data were presented as adjusted odds ratio (AOR) with 95% confidence intervals (CI). The level of significance was set at 0.05.

## Results

More than one-third of the participants whose EPDS scores were 13 or above were categorized as having PPD symptoms. Table 1 shows distributions of socio-demographic factors and their associations with PPD. Of 840 participants, most were aged between 18 and 30 (64.4%) and living with a partner (90.4%). Over 50% stayed in an extended family, resided in a rural area, lived in the South region, earned 15,000 THB (approximately 460 USD) or more monthly, and reported a food ‘secure’ condition (remained secure or improved). Bivariate analysis revealed that maternal age, education, marital status, residence, regions, monthly earnings, food insecurity status, delivery mode, intended pregnancy, number of children, birth interval, and health problems during the perinatal period were associated with PPD (*p* < 0.05).

**Table 1:** Sociodemographic factors by postpartum depression (PPD) among postpartum mothers in Thailand

Variables	*n*	(%)	PPD*				*p*-value
			No		Yes		
			*n*	(%)	*n*	(%)	
Total sample	840	(100.0)	568	(67.6)	272	(32.4)	
**Sociodemographic factors**							
Maternal Age (years)							
18–30	541	(64.4)	344	(63.6)	197	(36.4)	**< 0.001**
> 30	299	(35.6)	224	(74.9)	75	(25.1)	
Education							
Standard or no education	458	(54.5)	286	(62.5)	172	(37.6)	**< 0.001**
Higher education	382	(45.5)	282	(73.8)	100	(26.2)	
Employment							
Employed or self-employed	357	(42.5)	242	(67.8)	115	(32.2)	0.062
On (paid or unpaid) maternal leave	171	(20.4)	127	(74.3)	44	(25.7)	
Housewives or unemployed	312	(37.1)	199	(63.8)	113	(36.2)	
Marital status							
Married/living with a partner	759	(90.4)	531	(70.0)	228	(30.0)	**< 0.001**
Unmarried or not living with a partner	81	(9.6)	37	(45.7)	44	(54.3)	
Family types							
Extended	481	(57.3)	336	(69.9)	145	(30.2)	0.109
Non-extended	359	(42.7)	232	(64.6)	127	(35.4)	
Residential areas							
Rural	458	(54.5)	327	(71.4)	131	(28.6)	**0.010**
Urban	382	(45.5)	241	(63.1)	141	(36.9)	
Regions							
Central and Bangkok	308	(36.7)	183	(59.4)	125	(40.6)	**< 0.001**
North and Northeast	105	(12.5)	67	(63.8)	38	(36.2)	
South	427	(50.8)	318	(74.5)	109	(25.5)	
Monthly household income during COVID							
Less than 15,000 THB	404	(48.1)	251	(62.1)	153	(37.9)	**0.001**
15,000 or more THB	436	(51.9)	317	(72.7)	119	(27.3)	
Changes in food insecurity status							
No change (insecure to insecure)	236	(28.1)	130	(55.1)	106	(44.9)	**< 0.001**
Worse (secure to insecure)	180	(21.4)	121	(67.2)	59	(32.8)	
Secure (insecure to secure OR secure to secure)	424	(50.5)	317	(74.8)	107	(25.2)	
Infant age							
< 3 months of age	477	(56.8)	335	(59.0)	142	(52.2)	0.064
≥ 3 months of age	363	(43.2)	233	(41.0)	130	(47.8)	
Low infant birthweight							
Less than 2.5 kg	81	(9.6)	50	(61.7)	31	(38.3)	0.233
2.5 kg or higher	759	(90.4)	518	(68.3)	241	(31.8)	
Delivery mode							
Vaginal delivery	489	(58.2)	314	(64.2)	175	(35.8)	**0.013**
Caesarian section	351	(41.8)	254	(72.4)	97	(27.6)	
Intended pregnancy							
Yes	705	(83.9)	501	(71.1)	204	(28.9)	**< 0.001**
No	135	(16.1)	67	(49.6)	68	(50.4)	
Number of children							
One child	495	(58.9)	321	(64.9)	174	(35.2)	**0.040**
Two or more	345	(41.1)	247	(71.6)	98	(28.4)	
Birth interval							
No siblings	436	(51.9)	277	(63.5)	159	(36.5)	**0.017**
Less than 3 years	102	(12.1)	69	(67.7)	33	(32.4)	
3 years or higher	302	(36.0)	222	(73.5)	80	(26.5)	
Health problems during the perinatal period							
Yes	196	(23.3)	112	(57.1)	84	(42.9)	**< 0.001**
No	644	(76.7)	456	(70.8)	188	(29.2)	

Note: Bold text indicates a statistically significant difference with a p-value less than 0.05

* Mean score of PPD = 10.03

Table 2 shows distributions of infant feeding practices and their associations with PPD. Most of the participants breastfed their children directly (64.8%), and 59.9% fed expressed milk. Only a small proportion of participants fed their children with infant formula feeding (39%) and solid, semi-solid, or soft foods (7.9%). Infant feeding practices (direct breastfeeding, feeding expressed breastmilk, infant formula, complementary foods) and the impact of COVID-19 on breastfeeding practice (e.g., directly and expressed milk) were significantly associated with PPD in the bivariate analyses (*p* < 0.05).

**Table 2:** Infant feeding practices by postpartum depression (PPD) among postpartum mothers in Thailand

Variables	*n*	(%)	PPD				*p*-value
			No		Yes		
			*n*	(%)	*n*	(%)	
**Infant feeding practices last 24 h**							
Direct breastfeeding							
Yes	544	(64.8)	383	(70.4)	161	(29.6)	**0.019**
No	296	(35.2)	185	(62.5)	111	(37.5)	
Feeding expressed breastmilk							
Yes	503	(59.9)	327	(65.0)	176	(35.0)	**0.048**
No	337	(41.1)	241	(71.5)	96	(28.5)	
Infant formula feeding							
Yes	328	(39.0)	203	(61.9)	125	(38.1)	**0.005**
No	512	(61.0)	365	(71.3)	147	(28.7)	
Solid, semi-solid or soft foods							
Yes	66	(7.9)	24	(36.4)	42	(63.6)	**< 0.001**
No	774	(92.1)	544	(70.3)	230	(29.7)	
Infant feeding pattern							
Breastfeeding only	470	(56.0)	347	(73.8)	123	(26.2)	**< 0.001**
Mixed breastmilk and formula milk only	222	(26.4)	143	(64.4)	79	(35.6)	
Formula milk only	71	(8.5)	47	(66.2)	24	(33.8)	
Others	77	(9.2)	31	(40.3)	46	(59.7)	
**Timing of any solid**,** semi-solid**,** or soft foods**							
Not introduced yet	612	(72.9)	415	(73.1)	197	(72.4)	0.463
Before 6 months of age	36	(4.3)	21	(3.7)	15	(5.5)	
6 months of age or above	192	(22.9)	132	(23.2)	60	(22.1)	
**Impact of COVID-19 on breastfeeding practice**							
Direct breastfeeding							
Did/do not intend/shorter than intended	431	(51.3)	274	(63.6)	157	(36.4)	**0.010**
Same/longer than I intended	409	(48.7)	294	(71.9)	115	(28.1)	
Feeding expressed milk							
Did/do not intend/shorter than intended	415	(49.4)	263	(63.4)	152	(36.6)	**0.009**
Same/longer than I intended	425	(50.6)	305	(71.8)	120	(28.2)	

Note: Bold text indicates a statistically significant difference with a p-value less than 0.05

In Table 3, mothers’ responses to questions about their beliefs in COVID-19 vaccination were presented. Over half of the mothers (43.1%) agreed that “A COVID-19 vaccine is safe for breastfeeding women and babies”, while mothers who responded that they agreed or were undecided about “Breastfeeding women are not allowed to take a COVID-19 vaccine” and “Women should stop breastfeeding to take a COVID-19 vaccine” were 13.5% and 11.8%, respectively. 43.9% of mothers responded that they were worried about the side effects of the COVID-19 vaccine.

**Table 3:** Responses to each question about belief toward COVID-19 vaccination

	Strongly disagree (%)	Disagree (%)	Undecided (%)	Agree (%)	Strongly agree (%)
1) A COVID-19 vaccine is safe for breastfeeding women and baby	5.1	8.1	31.0	43.1	12.7
2) Breastfeeding women are not allowed to take a COVID-19 vaccine	26.2	24.9	32.4	13.5	3.1
3) Women should stop breastfeeding in order to take a COVID-19 vaccine	26.7	29.6	29.1	11.8	2.9
4) I am worried about potential side effects of a COVID-19 vaccine	7.9	12.6	22.7	43.9	12.9
Total score (4–20 scores)	13.32 ± 2.74				

Note: Q2-4 were reversely coded before summing

We further examined associations of beliefs towards COVID-19 vaccination and COVID-19-related attitudes and knowledge with types of infant feeding practices (Table 4). The postpartum mother feeding expressed milk to infants compared to the counterparts had significantly higher mean scores in COVID-19 related attitude, beliefs towards COVID-19 vaccination and COVID-19-related attitude and knowledge (*p* < 0.05). On the other hand, the mothers feeding infant formula or complementary foods had lower mean scores in beliefs towards COVID-19 vaccination, while those fed directly at the breast and complementary food had lower mean scores in COVID-19-related knowledge (*p* < 0.05).

**Table 4:** Mean (± SD) of mothers’ COVID-19-related attitude and knowledge and beliefs towards COVID vaccination by infant feeding practices

	Direct breastfeeding			Expressed breast milk			Infant formula			Solid, semi-solid or soft foods	
	No	Yes		No	Yes		No	Yes		No	Yes
	Mean (SD)	Mean (SD)		Mean (SD)	Mean (SD)		Mean (SD)	Mean (SD)		Mean (SD)	Mean (SD)
COVID-19 related attitude	28.5 (± 4.9)	28.5 (± 5.5)		27.9 (± 5.7)	28.9 (± 5.0)*		28.6 (± 5.4)	28.3 (± 5.2)		28.4 (± 5.3)	29.5 (± 5.5)
COVID-19 related knowledge	7.1 (± 1.7)	6.4 (± 2.2)**		6.4 (± 2.3)	6.9 (± 1.9)*		6.6 (± 2.2)	6.8 (± 1.9)		6.7 (± 2.1)	6.2 (± 2.1)*
Beliefs towards COVID-19 vaccination	13.2 (± 2.6)	13.4 (± 2.8)		13.0 (± 2.7)	13.6 (± 2.8)*		13.5 (± 2.8)	13.1 (± 2.7)*		13.4 (± 2.7)	12.7 (± 2.6)*

Note: *p < 0.05, **p < 0.0001

The distributions of COVID-19-related factors and their associations with PPD were presented in Table 5. Participants who had been ever diagnosed with COVID-19 positive were 17%, and those who had taken a COVID-19 vaccine were 55.8%. In light of the associations with PPD, while a history of prior COVID-19 infection and vaccination was not associated, COVID-19-related cognitive factors, such as beliefs about COVID-19 vaccination and COVID-19-related knowledge and attitude, were significantly associated (*p* < 0.05).

**Table 5:** Infant feeding practice and COVID-19 related factors by postpartum depression (PPD) in Thailand

Variables	*n*	(%)	PPD				*p* value
			No		Yes		
			*n*	(%)	*n*	(%)	
**COVID-19 related factors**							
Ever diagnosed as COVID-19 positive							
Yes	143	(17.0)	99	(69.2)	44	(30.8)	0.651
No	697	(83.0)	469	(67.3)	228	(32.7)	
Ever taken a COVID-19 vaccine							
Yes	469	(55.8)	308	(65.7)	161	(34.3)	0.175
No	371	(44.2)	260	(70.1)	111	(29.9)	
COVID-19-related attitude							
Negative (1st tertile)	236	(28.1)	151	(64.0)	85	(36.0)	**0.015**
Moderate (2nd tertile)	364	(43.3)	237	(65.1)	127	(34.9)	
Positive (3rd tertile)	240	(28.6)	180	(75.0)	60	(25.0)	
COVID-19-related knowledge							
Low (1st tertile)	321	(38.2)	219	(68.2)	102	(31.8)	**0.007**
Moderate (2nd tertile)	166	(19.8)	96	(57.8)	70	(42.2)	
High (3rd tertile)	353	(42.0)	253	(71.7)	100	(28.3)	
Beliefs about COVID-19 vaccination							
Low (1st tertile)	208	(24.8)	125	(60.1)	83	(39.9)	**0.017**
Moderate (2nd tertile)	252	(30.0)	171	(67.9)	81	(32.1)	
High (3rd tertile)	380	(45.2)	272	(71.6)	106	(26.4)	

Note: Bold text indicates a statistically significant difference with a p-value less than 0.05

Multiple logistic regression in Table 6 revealed that participants living in urban residency (AOR = 1.47; 95% CI = 1.02–2.05), residing in the Central Region and Bangkok (AOR = 2.16; 95% CI = 1.45–3.20) and the North and Northeast Region (AOR = 2.31; 95% CI = 1.34–3.99), encountering food-insecurity before and during COVID-19 (AOR = 0.56; 95% CI = 0.39–0.82), having unintended pregnancy (AOR = 1.58; 95% CI = 1.00–2.47), having health problems during perinatal period (AOR = 1.94; 95% CI = 1.30–2.89), feeding expressed breastmilk (AOR = 1.67; 95% CI = 0.95–2.90) or solid, semi-solid or soft foods (AOR = 3.84; 95% CI = 1.35–10.87), and COVID-related attitude (AOR = 1.91; 95% CI = 1.19–3.07 for a negative attitude and AOR = 1.85; 95% CI = 1.20–2.86 for a moderate attitude) had significantly higher odds of PPD.

**Table 6:** Adjusted odds ratios (AOR) and the 95% confidence intervals (CI) of postpartum depression (PPD) in Thailand

	PPD			
	AOR	(95% CI)		p value
Age (18–30 vs. >30 years)	1.24	(0.83-	1.85)	0.29
Education (No education or Standard vs. Higher education)	1.39	(0.92-	2.10)	1.22
Marital status	1.42	(0.71-	2.84)	0.35
Unmarried or not living with a partner vs. Married/living with a partner				
Residential area (Urban vs. Rural)	**1.47**	**(1.02-**	**2.05)**	**0.04**
Regions				
Central and Bangkok vs. South	**2.16**	**(1.45-**	**3.20)**	**< 0.001**
North and Northeast vs. South	**2.31**	**(1.34-**	**3.99)**	**0.05**
Monthly household income during COVID-19 pandemic				
<15,000 THB vs. ≥15,000 THB	1.42	(0.96-	2.10)	0.76
Changes in food insecurity status				
No change (insecure to insecure) vs. Secure (insecure/secure to secure)	**0.56**	**(0.39-**	**0.82)**	**< 0.05**
Worse (secure to insecure) vs. Secure (insecure/secure to secure)	0.44	(0.98-	1.93)	0.27
Delivery mode (Vaginal delivery vs. Caesarian section)	1.25	(0.86-	1.81)	0.25
Intended pregnancy (no)	**1.58**	**(1.00-**	**2.47)**	**< 0.05**
Number of siblings (singleton vs. ≥1 sibling)	0.60	(0.16	2.19)	0.44
Birth interval				
No sibling vs. 3 years or higher	1.13	(0.77-	1.67)	0.54
Less than 3 years vs. 3 years or higher	1.42	(0.81-	2.50)	0.22
Health problems during the perinatal period (yes)	**1.94**	**(1.30-**	**2.89)**	**< 0.001**
Direct breastfeeding (no)	1.18	(0.65-	2.15)	0.59
Feeding expressed milk (yes)	1.67	(0.95-	2.90)	0.07
Infant formula (yes)	1.19	(0.34-	4.10)	0.79
Solid, semi-solid or soft foods (yes)	**3.84**	**(1.35-**	**10.87)**	**0.01**
Infant feeding pattern				
Breastfeeding only vs. Others	0.98	(0.48-	1.98)	0.96
Mixed BF and formula vs. Others	0.77	(0.15-	3.88)	0.75
Formula only vs. Others	1.31	(0.36-	4.70)	0.68
Impact of COVID-19 on feeding on breast directly				
Did/do not intend/shorter vs. Same/longer	1.14	(0.77-	1.69)	0.52
Impact of COVID-19 on feeding expressed milk				
Did/do not intend/shorter vs. Same/longer	1.24	(0.83-	1.85)	0.29
COVID-19 related attitude				
Negative (1st tertile) vs. Positive (3rd tertile)	**1.91**	**(1.19-**	**3.07)**	**< 0.05**
Moderate (2nd tertile) vs. Positive (3rd tertile)	**1.85**	**(1.20-**	**2.86)**	**< 0.05**
COVID-19 related knowledge				
Low (1st tertile) vs. High (3rd tertile)	0.97	(0.64-	1.46)	0.88
Moderate (2nd tertile) vs. High (3rd tertile)	1.51	(0.95-	2.41)	0.08
Beliefs about COVID-19 vaccination				
Negative (1st tertile) vs. Positive (3rd tertile)	0.97	(0.63-	1.48)	0.88
Moderate (2nd tertile) vs. Positive (3rd tertile)	1.28	(0.80-	2.03)	0.30

Note: Bold text indicates a statistically significant difference with a p-value less than 0.05

## Discussion

This study revealed that one-third of the participants (32.4%) were at risk of having PPD. A recent review study showed PPD in Thailand ranged from 8.4–41.7% [23]. The differences are due to different selection criteria used (e.g., postpartum period, sample size, and setting), application of various screening tools and cutoff points, and context-specific (i.e., during the COVID-19 pandemic).

Despite the benefits of COVID-19 vaccines, vaccination of lactating women remains a public health issue because of the notion that vaccines may affect breast milk. Nonetheless, data on maternal beliefs about COVID-19 vaccination are very limited. In this present study, approximately 8.1% of mothers disagreed that “the COVID-19 vaccine is safe for breastfeeding women and babies,” while 43.1% agreed and 31.0% were undecided in our study. A study at a university hospital in Bangkok, Thailand [18] reported that the proportion of breastfeeding mothers who said “vaccines cause harm to you” or “vaccines cause harm to newborns” were 39.5% and 22.8%, respectively. Regarding the vaccination while breastfeeding, 35% of mothers agreed that “Breastfeeding mothers should not be vaccinated while breastfeeding” [18]. This is higher than the response rates in our study, where 13.5% and 11.8% agreed with the statement “Women should stop breastfeeding to take a COVID-19 vaccine” and “Breastfeeding women are not allowed to take a COVID-19 vaccine,” respectively. The differences in response to the vaccine misperception may be due to the socioeconomic characteristics of the study population, such as education and study area. In our study, 45.5% of mothers recruited nationwide, including Bangkok, had a bachelor’s degree or higher, while 52.8% of mothers recruited from a large university hospital in Bangkok had a bachelor’s degree or higher.

We further examined the association of cognitive factors, such as belief towards COVID-19 vaccination and COVID-19-related knowledge and attitude with infant feeding practices. To our knowledge, our study is the first to show the associations of belief towards COVID-19 vaccination with types of infant feeding practice. Our bivariate analysis revealed that postpartum mothers feeding expressed milk to their infants had positive beliefs towards breastfeeding and COVID-19 vaccination and higher scores of COVID-19 knowledge and attitude, whereas the mothers who fed their infants with infant formula, or solid, semi-solid, or soft food are likely to have negative beliefs towards COVID-19 vaccination. Wang et al. [20] showed that COVID-19-vaccinated mothers breastfed longer and had higher COVID-19 knowledge, attitude, and behavior scores than their counterparts (*p* < 0.01). Some studies found that poor attitudes towards COVID-19 are related to exposure to unreliable or misinforming sources of information [12, 24], which can cause more stress to mothers due to anxiety and the inability to make the correct decision to raise their infants while preventing COVID-19. A cross-sectional study in Saudi Arabia investigating attitudes and fears regarding COVID-19 vaccination among pregnant and breastfeeding women reported no association between COVID-19 vaccine acceptance and breastfeeding [25]. The findings of this study may suggest that with the protective effects of breastfeeding on mothers and babies, greater efforts should be made to breastfeeding education and public campaigns to increase mothers’ belief towards breastfeeding and vaccination through providing good quality information on the benefits of breastfeeding against emerging infectious diseases such as COVID-19. Further research is needed to determine how these beliefs affect the association between feeding methods and PPD. Meanwhile, there was no association between the COVID-19 vaccine uptake and PPD in our study. However, a Chinese study reported that those with higher depression scores were more likely to skip the vaccination [20].

Our study also has shown that postpartum mothers who fed their infants with solid, semi-solid, or soft foods were more likely to have PPD. Similar results were reported in a pooled sample of multinational studies from which the present study sample was taken, with significantly higher odds of reporting PPD among postpartum mothers who were fed solid, semi-solid, or soft foods [26]. A scoping review exploring breastfeeding practices during the COVID-19 pandemic in low-, middle- and high-income countries reported that COVID-19-related measures that isolate the mothers from their infants (e.g., quarantine during hospitalization after giving birth or being called back to work onsite) affect maternal breastfeeding behavior [27]. As COVID-19 measures increased food prices, including infant formula, and reduced the availability of infant food, mothers may become dependent on less expensive food sources for their infants [28]. It may explain the reason why postpartum mothers who fed their infants with infant formula or complementary food were more likely to be classified as PPD [27]. Additionally, the time and difficulty in preparing baby food can also make mothers more depressed [29]. In particular, employed mothers shouldered double the burden of child care and work life, increasing their stress, especially in families where fathers cannot share child care. Support for postpartum mothers does not only rest with the public health workers or professionals but also their partners. Family support, especially from a spouse, is crucial in alleviating stress related to reducing the chance of developing PPD.

In our study, some sociodemographic factors significantly associated with a mother’s PPD include food insecurity before and during the COVID-19 pandemic, urban residency setting, living in Bangkok, Central, North or Northeast regions, unintended pregnancy, and health problems during the perinatal period. The postpartum women experiencing food insecurity during the pandemic reported having experienced PPD symptoms. Lack of food as a fundamental factor for survival exacerbated the stress on the mothers [30–32]. In addition, during the pandemic, the situation could be worsened as COVID-19 prevention and control measures could hinder the transportation of food supplies between cities, thus reducing accessibility and availability of food items in some areas [8]. Moreover, the implemented measures forced some businesses to close down and lay off their staff, including postpartum mothers. Therefore, the unemployed mothers encountered difficulty in earning enough money to buy food for their infants and themselves [33]. In particular, living in an urban area poses a great challenge due to the dynamic of COVID-19 prevention and control regulations, which restrict personal contact between mothers and their peers or relatives [8]. Moreover, the implemented measures forced some businesses to close down and lay off their staff, including postpartum mothers. Therefore, the mothers with low-income encountered difficulty in earning enough money to buy food for their infants and themselves [28], which contributed to the risk of PPD. In addition, our study showed that mothers who lived in Bangkok, Central, North, or Northeast Thailand are at higher risk of having PPD-related symptoms compared to their Southern counterparts. Central (including Bangkok), North, and Northeast of Thailand were heavily enforced by COVID-19 prevention and control measures [8]. Restriction measures led to business shut down and staff lay-off across Thailand. The most impacted groups were vulnerable individuals, including postpartum mothers, who lived in urban areas. Many studies have shown that postpartum mothers living in urban areas are more likely to experience postpartum depression than postpartum mothers living in rural areas [34, 35]. In addition, women who had no intention to get pregnant and health problems during the perinatal period were associated with a higher probability of having PPD-related symptoms. Previous studies also reported that postpartum women who had no intended pregnancy encountered a higher level of depression compared to their experienced peers [34]. Health problems during pregnancy were also reported in many studies as the factors that exacerbated stress or depression after delivery [36–38]. Although some socio-demographic variables, such as maternal age and education, marital status, and monthly household income, did not remain significant in the multiple logistic regression, these factors are important when designing programs or interventions for the prevention and early detection of PPD. Since young mothers experienced hardship in child rearing both before and during the COVID-19 pandemic, sufficient consultancy and physical support to support child-rearing among the young and living-alone mothers is imperative [26, 39, 40].

Though we found an association between PPD and the associated factors, causal relationships cannot be established due to the cross-sectional study design. In addition, the mothers in the study included those with Internet accessibility and infants up to six months old using a convenience sampling technique. Consequently, the findings might not be representative of all Thai postpartum women. A longitudinal study design is recommended to determine the causal relationships between the interest factors. This study did not ask about the number and timing of COVID-19 vaccinations, which may affect belief in the COVID-19 vaccine. Lastly, we did not examine the direct or indirect impact of infant feeding behaviors or cognitive factors on PPD. Therefore, caution is needed when interpreting this. Since there is no evidence supporting the belief that COVID-19 could be transmitted through breastmilk, health, and non-health authorities should encourage breastfeeding among women who were or are diagnosed as COVID-19 positive, along with advice to wear facial protection while breastfeeding to reduce the infant’s exposure to droplets from the mother.

## Conclusion

Our study found that COVID-19-related cognitive factors and infant feeding practices were associated with postpartum depression. This suggests that providing postpartum mothers with education on preventing and managing diseases such as COVID-19 and on appropriate infant feeding practices may ultimately help improve maternal mental health.

## Data Availability

All data generated or analyzed during this study are included in this article.

## Abbreviations

AOR: Adjusted odds ratio
CI: Confidence interval
COVID-19: Coronavirus disease 19
PPD: Postpartum depression
WHO: World Health Organization
